# Molecular characterization of rotavirus isolates from select Canadian pediatric hospitals

**DOI:** 10.1186/1471-2334-12-306

**Published:** 2012-11-15

**Authors:** Andrew McDermid, Nicole Le Saux, Elsie Grudeski, Julie A Bettinger, Kathy Manguiat, Scott A Halperin, Lily MacDonald, Pierre Déry, Joanne Embree, Wendy Vaudry, Timothy F Booth

**Affiliations:** 1 National Microbiology Laboratory, Winnipeg, Canada; 2 Department of Medical Microbiology, University of Manitoba, Winnipeg, Canada; 3 Division of Infectious Diseases, CHEO Research Institute, Children’s Hospital of Eastern Ontario, Ottawa, Canada; 4 Vaccine Evaluation Centre, BC Children’s Hospital, University of British Columbia, Vancouver, Canada; 5 Canadian Center for Vaccinology, IWK Health Centre and Dalhousie University, Halifax, Canada; 6 Centre Mere-Enfant de Quebec, CHUL, and Laval University, Quebec City, Canada; 7 Winnipeg Children's Hospital, Winnipeg, Canada; 8 Stollery Children’s Hospital and University of Alberta, Edmonton, Canada

## Abstract

**Background:**

We report the first multi-site rotavirus genotype analysis in Canada. Prior to this study, there was a dearth of rotavirus G and P genotyping data in Canada. Publically funded universal rotavirus vaccination in Canada started in 2011 and has been introduced by four provinces to date. Uptake of rotavirus vaccines in Canada prior to 2012 has been very limited. The aim of this study was to describe the genotypes of rotavirus strains circulating in Canada prior to widespread implementation of rotavirus vaccine by genotyping samples collected from selected paediatric hospitals. Secondly we identified rotavirus strains that differed genetically from those included in the vaccines and which could affect vaccine effectiveness.

**Methods:**

Stool specimens were collected by opportunity sampling of children with gastroenteritis who presented to emergency departments. Samples were genotyped for G (VP7) genotypes and P (VP4) genotypes by hemi-nested multiplex PCR methods. Phylogenetic analysis was carried out on Canadian G9 strains to investigate their relationship to G9 strains that have circulated in other regions of the world.

**Results:**

348 samples were collected, of which 259 samples were rotavirus positive and genotyped. There were 34 rotavirus antigen immunoassay negative samples genotyped using PCR-based methods. Over the four rotavirus seasons, 174 samples were G1P[8], 45 were G3P[8], 22 were G2P[4], 13 were G9P[8], 3 were G4P[8] and 2 were G9P[4]. Sequence analysis showed that all Canadian G9 isolates are within lineage III.

**Conclusions:**

Although a limited number of samples were obtained from a median of 4 centres during the 4 years of the study, it appears that currently approved rotavirus vaccines are well matched to the rotavirus genotypes identified at these hospitals. Further surveillance to monitor the emergence of rotavirus genotypes in Canada is warranted.

## Background

Group A rotaviruses are a major cause of acute gastroenteritis in children under five, causing an estimated 453,000 deaths worldwide annually [1,2]. In Canada, as in other developed countries, mortality is rare, although rotavirus infections are costly from both societal and health care system perspectives. Recently published research on children with gastroenteritis admitted to Canadian paediatric hospitals found that rotavirus infections require 1300–1800 tertiary care hospital days annually [3]. Furthermore, rotavirus gastroenteritis results in outpatient visits and nonmedical costs, such as lost work days that have a substantial economic cost [3-6].

The outer capsid of rotavirus is composed of two major antigenic proteins, VP4 and VP7. These proteins are the main determinants of viral serotype, and the genes that code for these proteins represent the P and G genotype of rotaviruses respectively. The VP4 and VP7 proteins are also the main targets for protective neutralizing antibodies, and are thus key antigens in vaccine development [7-9]. To date, 27 G genotypes and 35 P genotypes have been reported, although only 11 G genotypes and 12 P genotypes have been recovered from humans [10-14]. G and P genotypes are also 2 components of the 11-component genotyping system proposed to classify rotaviruses [14]. Five combinations of these genotypes, G1P[8], G2P[4], G3P[8], G4P[8] and G9P[8] are responsible for the majority of rotavirus infections worldwide. These are also the most common genotypes in North America, where they represent more than 85% of rotaviruses detected in human gastrointestinal disease [12,15-18]. Studies that have analysed only the G genotypes, also found that G1, G2, G3, G4 and G9 predominated in North America [19-21].

Two live oral rotavirus vaccines were licensed in Canada. RotaTeq®, licensed in Canada in August 2006, is a pentavalent bovine reassortant vaccine based on the bovine rotavirus WC3 strain as a backbone, and each of the 5 vaccine strains contains one serotype of the human outer capsid proteins (G1, G2, G3, G4, or P[8] serotypes). Rotarix®, licensed in Canada in July 2007, is a monovalent live-attenuated G1P[8]vaccine derived from human rotavirus 89–12, containing the two most common outer capsid serotypes. Extensive phase III trials for these vaccines showed high efficacy in protecting children against rotavirus disease of any severity, for strains with the same serotypes as contained in the respective vaccine, and there was a significant degree of cross-reactivity against many genotypes not contained in the vaccines [22]. Vaccines have been shown to be very effective in the United States, although changes in antigenic properties of circulating strains, due to antigenic drift or recombination, may challenge the effectiveness of the vaccines in the future [23-28]. In 2008, the National Advisory Committee for Immunization (NACI) and the Canadian Paediatric Society recommended the use of rotavirus vaccine in Canada [29,30]. Despite this, the uptake of rotavirus vaccines in Canada has been very limited. Prior to this study, there was very little data on the incidence of rotavirus G and P genotypes in Canada. During the period of this study, vaccination use in Canada was very low, well below 5% of the eligible population were getting rotavirus vaccines (personal observations). Starting in 2012, only four of 13 provinces and territories offered rotavirus vaccine as part of their publicly funded immunization programs [31].

To survey the baseline prevalence of rotavirus genotypes prior to the introduction of rotavirus vaccine we collected specimens from inpatients at select paediatric hospitals. We utilised reverse transcriptase polymerase chain reaction (RT-PCR) followed by hemi-nested multiplex polymerase chain reaction methods for P and G genotyping [13,28,32-34]. Selected rotavirus strains were further characterised by phylogenetic analysis: in particular we focussed on the Canadian G9 strains, since this genotype has emerged and spread worldwide in recent years, and is not a specific component of either of the current vaccines.

## Methods

A total of 348 stool samples were collected from children that presented with diarrhea with or without vomiting to eight Canadian pediatric hospital emergency departments that were part of the Canadian Immunization Monitoring Program, Active (IMPACT) network [35] from 2007 to 2010. Five pediatric hospitals were included in the study during the first year (2007: Edmonton, Winnipeg, Ottawa, Quebec City, Halifax), three hospitals for the years 2008 and 2009 (Ottawa, Quebec City, Halifax) and six in 2010 (Vancouver, Saskatoon, Winnipeg, Toronto, Ottawa, Halifax). The total number of specimens collected at the sites were as follows: Ottawa, 128; Halifax, 90; Quebec City, 63; Edmonton, 24; Vancouver, 20; Saskatoon, 11; Winnipeg, 9; Toronto, 3.

The study was approved by the research ethics boards of each hospital, in accordance with the Helsinki Declaration on ethical principles for medical research involving human subjects. Specimens were collected from children under 5 years of age that presented with diarrhea with or without vomiting at emergency departments during 2007, 2008 and 2009 [6]. Research nurses at the emergency department of each centre approached parents or guardians of children presenting with acute diarrhea and asked them to consent to rotavirus testing on the child’s stool sample. In 2010 the samples were from children under 16 years of age who were hospitalized for laboratory confirmed rotavirus infection at six hospitals (Vancouver, Saskatoon, Winnipeg, Toronto, Ottawa, Halifax).

At each hospital stool samples underwent testing for rotavirus by enzyme immunoassay (Premier rotaclone EIA kit, Meridian Bioscience), chromatographic immunoassay (bioMerieux Vikia Rota-Adeno, or Coris Bioconcept Combi strip) or by electron microscopy (EM). All specimens were then sent to the National Microbiology Laboratory for further testing and genotyping.

Genomic RNA was extracted from the stool samples using a Nuclisens Easymag magnetic silica extraction method (Biomerieux, France). Hemi-nested multiplex PCR assays were used to P and G genotype the extracted RNA [32,36-40]. PCR products were initially designated to genotype based on size comparison after direct visualization after electropheresis on agarose gels. During the course of genotyping work we replaced a previously described primer specific for P[8] genotypes, 1T-1 [36], with the degenerate primer 1T-1DCDN since it was found to be complementary to a wider range of P[8] strains. The 1T-1DCDN primer was designed with the sequence 5’ TCT ACT GGR TYR ACR TG 3’ using the primer 1T-1D [38] as a model, and an alignment of the VP4 variable region using the Clustal W algorithm in MEGA 5.0 [41]. The alignment included sequences from 66 previously untypable Canadian P[8] samples (determined by genetic distance after sequencing), one Canadian P[4] sample, and 15 P[8] reference strains. The 1T-1DCDN primer binds at nt 340–356, a similar position, but one nucleotide shorter than that of 1T-1 (nt 339–356).

The new 1T-1DCDN primer was compared with 1T-1 primer for genotyping using a panel of 65 Canadian rotavirus samples that included 47 samples that were sequence-positive for P[8], 4 specimens that were P[4] genotype and 14 that were rotavirus negative. The panel was representative in that it was composed of specimens that came from 5 different sites in 2007, 3 sites in 2008 and 2 sites in 2009.

All strains that were positively genotyped by hemi-nested multiplex PCR were confirmed by sequencing of the VP4 and VP7 regions, using the Con3/Con2 and Beg9/End9 primer sets, respectively [36,37], or a suitable alternative. PCR products were purified using Montage PCR Centrifugal Filter Devices (Millipore, USA) and then cloned for sequencing in Top10 chemically competent *E. coli* cells (Invitrogen, USA) using Invitrogen 5’TopoTA cloning kits or sequenced directly. Sequencing of plasmids was carried out by the Genomics Core section of the National Microbiology Laboratory using T3 and T7 plasmid-specific primers.

Phylogenetic analysis was used for confirmation of genotypes and for further analysis of the ORFs of Canadian G9 strains. The Canadian G9 ORF sequences of the strains RVA/Human-wt/CAN/RT034-07/2007/G9P[8] through to RVA/Human-wt/CAN/RT088-09/2009/G9P[8] (GenBank Accession numbers JF964998-JF965010) were aligned, along with reference strains. Phylogenetic trees were constructed using the Clustal W algorithm in MEGA 5.0 software package [41], using the Maximum Likelihood method for phylogenetic analysis, with 1000 bootstrap replicates. Lineage designation was based on similarity to previously defined lineage reference strains [42,43].

## Results

**Figure 1:**
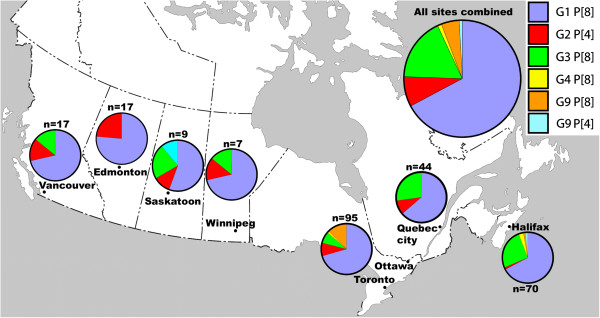
**Map showing the 8 study sites in Canada (black dots).** Above each site is a pie chart showing the relative prevalence of rotavirus genotypes detected between the years 2007–2010. The limited Toronto data (available for 2010 only) is included with Ottawa. A pie chart to the top right of the map indicates the cumulative prevalence of all genotypes in the study for all the years of study.

Stool samples were collected from children with gastroenteritis at eight different Canadian hospital sites between 2007 and 2010 (Figure 1), although only two of the sites were sampled in all four years, and three sites were sampled in only one year. Of 348 specimens, 271 were genotyped. Amongst these, 12 samples exhibited multiple genotypes, suggestive of mixed infections. Therefore, 259 specimens (74%) were assigned to a single G and P genotype (Table 1) and 77 were negative.

**Table 1:** Rotavirus identifications shown by genotype, year and surveillance site

**City**	**Year**	**Negative**	**G1P[8]**	**G2P[4]**	**G3P[8]**	**G4P[8]**	**G9P[8]**	**G9P[4]**	**Mixed**
Vancouver	2010	3	9	3	5	0	0	0	0
Edmonton	2007	1	13	4	0	0	0	0	6
Saskatoon	2010	2	5	1	2	0	0	1	0
Winnipeg	2007	1	3	0	0	0	0	0	0
	2010	1	2	1	1	0	0	0	0
Toronto	2010	0	3	0	0	0	0	0	0
Ottawa	2007	2	25	1	1	1	0	0	0
	2008	27	16	3	4	0	0	0	1
	2009	4	20	3	0	0	12	0	1
	2010	1	3	1	2	0	0	0	0
Quebec City	2007	4	15	1	7	0	0	0	0
	2008	12	7	1	0	0	0	0	0
	2009	1	6	2	5	0	0	0	2
Halifax	2007	1	5	1	1	0	1	0	1
	2008	5	29	0	0	0	0	0	0
	2009	12	3	0	17	2	0	0	1
	2010	0	10	0	0	0	0	1	0
Total		77	174	22	45	3	13	2	12

Of the 348 samples that were screened for rotavirus in hospital, 246 (71%) were positive by antigen immunoassay, and 102 (29%) screened negative. Of the specimens that had screened positive in hospital 237 (96%) were positively P and G for genotyped by PCR. Of the 102 specimens that had screened negative by antigen immunoassay, 34 (33%) were also successfully P and G genotyped for rotavirus. Twelve (4.4%) of the rotavirus-positive samples were indeterminate since they were apparently mixed infections with samples containing two or more genotypes of rotavirus (Table 1). The mixed samples included three instances of G1P[4] + P[8] , six instances of G1P[8] + G2P[4] and one instance each of G1 + G3P[8], G3P[4] + P[8] and G1 + G9P[8]. Of the 57 stool samples from 2010 that were positive locally for rotavirus, 50 were successfully genotyped and confirmed by sequencing.

**Figure 2:**
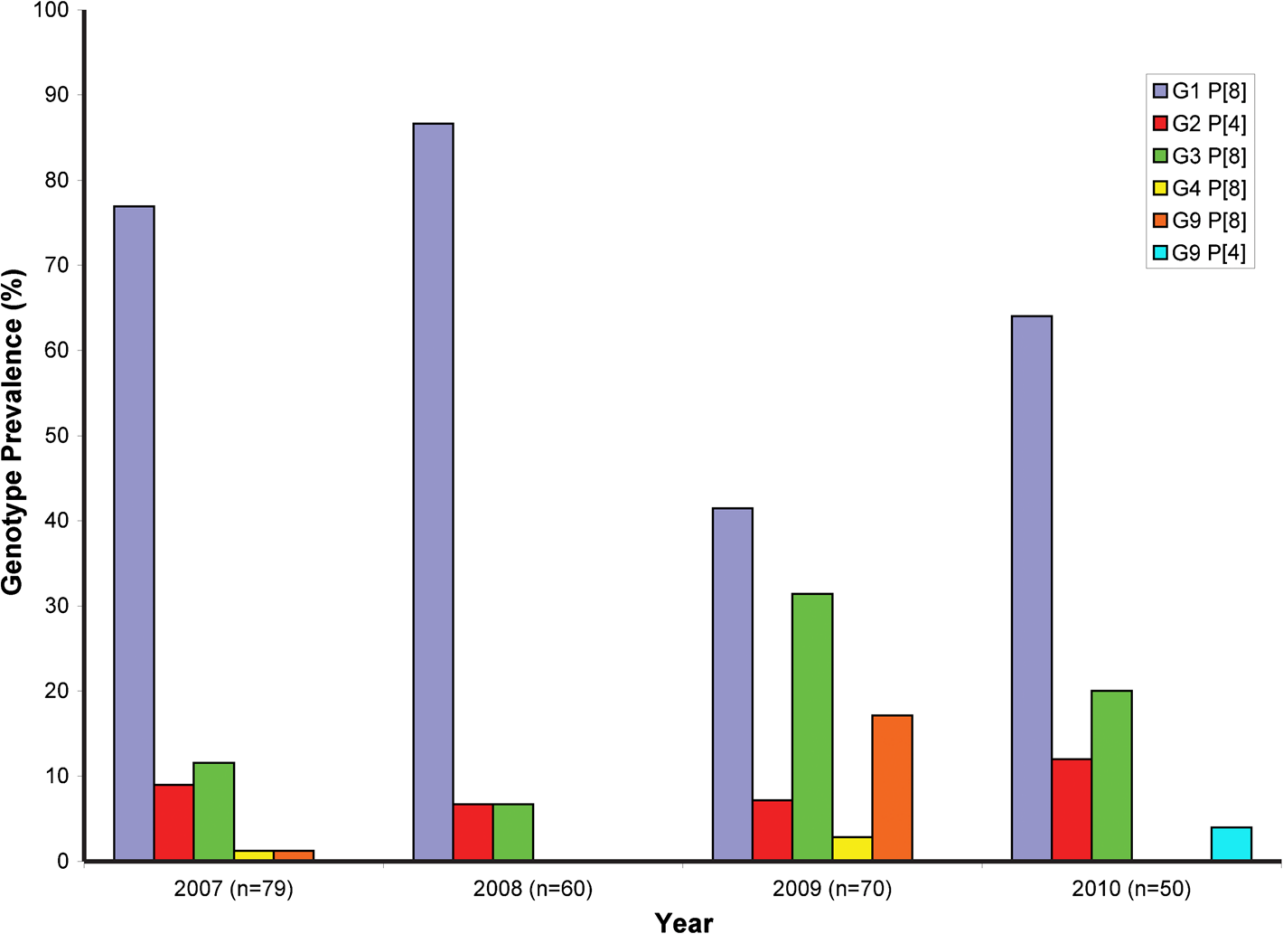
**Genotype prevalence by year for all Canadian sites.** The four seasons of surveillance are represented from left to right.

Overall, the most common genotype encountered in this study was G1P[8], being present in 67% of the specimens, and it was also the most prevalent genotype at all sites (Figure 1) and during all 4 years (Figure 2). The other common genotypes were G3P[8] (18%) and G2 P[4] (8.5%). At one site (Ottawa) during 2009 there was a significantly higher incidence of G9P[8] and a lower proportion of G1P[8] genotypes (Table 1; 95% CI, modified Wald method for proportions).

With the P[8] genotyping assay, we found that all 31 of the false negatives from the 1T-1 primer were picked up as P[8] samples by the 1T-1DCDN primer. In this panel (which was deliberately weighted with samples that were not picked up by the 1T-1 primer), the 1T-1DCDN primer had a sensitivity of 98% and a specificity of 90%, whereas the 1T-1 primer had a sensitivity of only 60% and a specificity of 82%. Thus 1T-1DCDN can be usefully added to the panel for genotype screening tests, and could potentially replace the 1T-1 primer.

**Figure 3:**
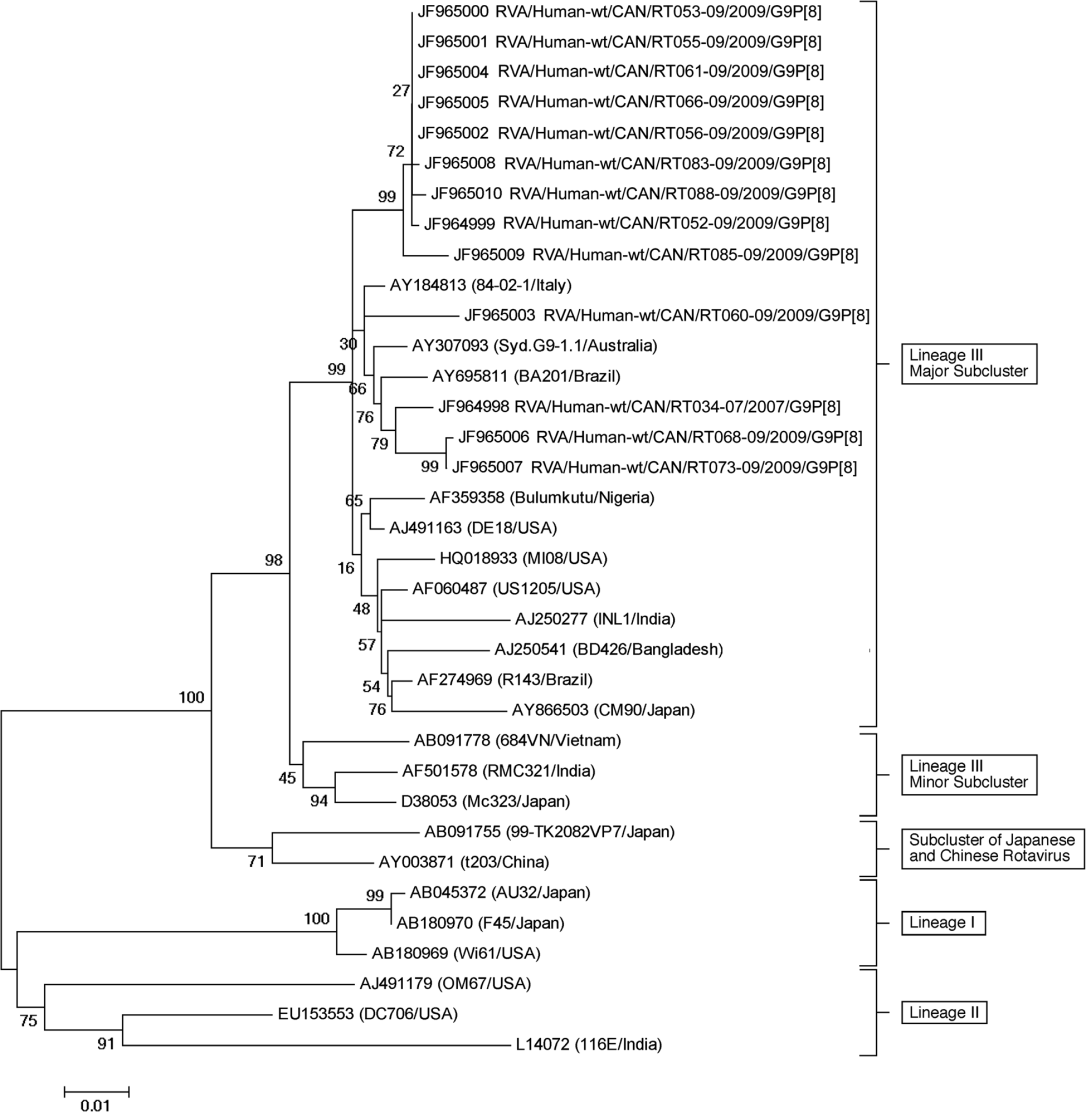
**Phylogenetic tree of the VP7 genome segments of Canadian isolates of rotavirus along with selected G9 prototype sequences.** The maximum-likelihood tree was bootstrapped 1000 times. Canadian G9P[8] strains are represented in the tree. All Canadian strains are designated with “RT” and their numbers end with the year of specimen collection.

A phylogenetic tree constructed with full VP7 gene sequences from all of the Canadian G9P[8] isolates including several prototype G9 sequences from different regions of the world is shown in Figure 3. The tree shows that the Canadian G9 isolates fall into the lineage III major subcluster, being more similar to G9 sequences from isolates obtained globally than to previously sequenced minor subcluster lineage III isolates from Asia.

## Discussion

Here we report the results of the first systematic, multi-site and multi-season rotavirus genotyping study in Canada. The most prevalent genotype found in children with gastroenteritis at all the sites throughout the four years of the study was G1P[8]. Other common genotypes were G3P[8] and G2P[4]. Until the present study, only two previous Canadian genotyping studies had been carried out: these were restricted to G-genotyping only at single sites during single seasons [19,21]. Our current genotyping data indicate that the available vaccines should be serologically well-matched to the prevalent strains of rotavirus, based on the genotype prevalence at these sites. Previous single-site studies in Canada during 1999 and 2002 also found G1 as the most prevalent rotavirus genotype [19,21]. Although G1, G2, G3 and G4 are the main serotypes present in North America and Europe, other serotypes are important causes of rotavirus gastroenteritis worldwide, such as G5 in South America and G8 in Africa [44-49]. G9 strains emerged in the 1990s as a cause of a significant proportion of rotavirus cases which varied regionally from 5 to 90% [44,50-53]. Although we did not sample every site in every year in the current study, our results provide recent data on the occurrence of rotavirus genotypes in Canada, during the period prior to vaccine roll-out. Our sampling rate is equivalent to about 1 per 100,000 population over the 4 years of the study in Canada, covering a median of 4 centres. This sampling rate compares favourably with other surveillance studies including one carried out previously in the United States [48]. We calculate that our study would have a 95% probability of detecting a rotavirus genotype with an incidence of 1.5% per year. Therefore, this level of sampling may miss any rare genotypes that occur incidentally and which do not spread in the population beyond one to two percent. G9P[8] strains were identified in the current study but were mostly restricted to single isolates in a few sites during 2 seasons (2007 and 2010) rather than fully emerging as a prevalent genotype. Recently, G9 genotypes have also been identified in the United States and comprised 39% of the rotavirus genotypes indentified in Detroit between 2007 and 2009 [41]. Since G9 was only substantially present in one site and for one season in Canada (Ottawa in 2009), and could therefore be a genotype that occurs with irregular frequency in Canada, we decided to investigate the possible origins of these strains using phylogenetic analysis. This shows that the Canadian G9 rotavirus strains collected in the present study are part of G9 lineage III (Figure 3). Although up to six lineages of G9 have been described, most viruses fall into three main lineages, I, II and III: a small number of G9 strains that do not fit into these lineages have also been reported [43]. The G9 genotype emerged worldwide starting in about 1995, and appeared to spread worldwide during the 2000s [43]. Sequence analysis indicated that one particular subcluster of G9 lineage III seemed to spread and cause disease throughout much of the world [43]. Most of the circulating G9 rotaviruses worldwide, including all of the G9 specimens from our study, are in the major subcluster of lineage III [43]. Thus Canada can be added to the growing list of countries where this lineage has been identified. Lineage I and II G9 rotaviruses are less common and were detected primarily within the United States, Japan and India in the 1980s and 90s, but reports of new lineages circulating in other countries underline the importance of continued rotavirus surveillance. [12,46,54-56].

Based on sequencing of a 981 bp region of the open reading frame of VP7, the Canadian G9 strains are more similar to isolates from Australia, Brazil and Italy than the G9 strain found in Detroit designated MI08/USA (Figure 3). Sequencing data from the two Canadian 2010 G9 strains also showed that they have between 96 and 98.5 percent identity with 2009 Canadian rotavirus strains. In addition the 2010 strains are most similar to the 2007 G9P[8] strain from Halifax, rather than the 2009 Ottawa strains (data not shown). Although G9 is not present in either vaccine, there is serological cross protection amongst G genotypes and amongst differing P genotypes since the majority of these Canadian G9 viruses were G9P[8] and the P[8] antigenic component is present in both vaccines. Given that two G9P[4] strains were collected in 2010, surveillance of genotype data is warranted to monitor genotype prevalence as there is the possibility of an increase in the prevalence of rarer genotypes, or genetic drift leading to immune escape [28,44]. For example, emerging strains such as G12 have been detected in New York State [57] and in Italy [58] and may spread to Canada in the future.

The local fluctuation of genotype prevalence that is frequently seen from year to year in circulating rotavirus was also demonstrated by less frequent genotypes such as G3P[8], that was more prevalent in 2009 and accounted for 77% of all rotavirus-positive samples from Halifax. The high prevalence of G3P[8] in Halifax in 2009 was preceded and followed by seasons in which no G3P[8] samples were detected at that site.

## Conclusions

Hemi-nested multiplex PCR is a rapid method for genotyping rotavirus samples, based on highly conserved genotype-specific regions [32,36,37]. Nevertheless, mutations may cause mispairing resulting in an untypable strain or a mistyping error [39]. Therefore we monitored the primer-binding regions of isolates collected during the study for genetic differences that could affect sensitivity and specificity. The 1T-1DCDN primer was developed to genotype Canadian P[8] samples that have mismatches in the binding region of the previously used 1T-1 primer, which failed to genotype many of the Canadian P[8] isolates. The 1T-1DCDN primer was validated for specificity and sensitivity for genotyping unidentified Canadian P[8] samples as well as those previously identified with 1T-1, and thus may be useful in future Canadian genotyping studies.

The finding of rotavirus in one third of samples which were negative by antigen testing or EM in this study also illustrates the lower sensitivity of antigen testing for rotavirus. However, antigen detection tests may be less prone to detecting low level background infections, and are therefore useful for studies to measure the burden of rotavirus illness, as well as for investigating the effectiveness of vaccine in decreasing all-cause gastrointestinal illness in the younger age groups. Our findings in this study strongly suggest that currently licensed vaccines are well matched to the rotavirus strains present in recent years in Canada, and that continued surveillance is warranted to monitor the situation after Canadian universal vaccination programs have been fully introduced.

## Competing interests

There were no competing interests for the authors of this study.

## Authors’ contributions

AM: carried out the molecular genetic studies, sequence analysis and drafted the manuscript. NL: Contributed to conception and study design and provided critical review of manuscript. EG: Carried out molecular genetic studies, sequence analysis. Supervised laboratory work and training. Involved in analysis and interpretation of data. JAB: Contributed to conception and study design and provided critical review of manuscript. KM: carried out molecular genetic studies, sequence analysis. SAH: Contributed to conception and study design and provided critical review of manuscript. LM: Carried out molecular genetic studies, sequence analysis. PD: Contributed to conception and study design and provided critical review of manuscript. JE: Contributed to conception and study design and provided critical review of manuscript. WV: Contributed to conception and study design and provided critical review of manuscript. TFB: Principal investigator; Supervised laboratory work. Involved in design of laboratory tests and analysis and interpretation of data. Contributed to conception and study design. Writing of final versions of the manuscript. All authors read and approved the final manuscript.

## Pre-publication history

The pre-publication history for this paper can be accessed here:

http://www.biomedcentral.com/1471-2334/12/306/prepub
